# Anticoagulant Property of a Sulfated Polysaccharide with Unique Structural Characteristics from the Green Alga *Chaetomorpha aerea*

**DOI:** 10.3390/md21020088

**Published:** 2023-01-26

**Authors:** Ling Qin, Yajing Yang, Wenjun Mao

**Affiliations:** 1 Key Laboratory of Marine Drugs of Ministry of Education, Shandong Provincial Key Laboratory of Glycoscience and Glycotechnology, School of Medicine and Pharmacy, Ocean University of China, Qingdao 266003, China; 2 Laboratory for Marine Drugs and Bioproducts, National Laboratory for Marine Science and Technology (Qingdao), Qingdao 266237, China

**Keywords:** sulfated polysaccharide, structural characteristics, anticoagulant activity, coagulation factor

## Abstract

Sulfated polysaccharides from marine algae have attracted a great amount of attentions for the development of marine drugs due to their unique structural features, and they are great potential sources of naturally occurring anticoagulant agents. The genus *Chaetomorpha* is one of the largest genera in green algae and has a worldwide distribution. In the present study, a homogeneous polysaccharide from *Chaetomorpha aerea*, designated as PCA, was obtained by alkali extraction, anion-exchange and size-exclusion chromatography. Based on the results of chemical and spectroscopic analyses, PCA was a sulfated galactoarabinan which was mainly constituted of a backbone of →4)-β-l-Ara*p*-(1→ unit, partially sulfated at C-3 of →4)-β-l-Ara*p*-(1→ and C-4 of →6)-α-d-Gal*p*-(1→. The side chains consisting of →6)-α-d-Gal*p*-(1→ and →5)-α-l-Ara*f*-(1→ residues were in C-2 of →4)-β-l-Ara*p*-(1→ unit. PCA had a strong anticoagulant activity in vitro as evaluated by the assays of activated partial thromboplastin time, thrombin time and fibrinogen level. The obvious anticoagulant activity in vivo of PCA was also found. PCA significantly inhibited the activities of the intrinsic coagulation factors XII, XI, IX and VIII, and exhibited weak inhibition effects on the common coagulation factors II and X. The anticoagulant mechanism of PCA was attributed to strong thrombin inhibition potentiated by heparin cofactor II or antithrombin III, and it also possessed an apparent inhibition effect on coagulation factor Xa mediated by antithrombin III. The investigation demonstrated that PCA could be a promising anticoagulant agent for health promotion and the treatment of thrombotic diseases.

## 1. Introduction

Thrombosis is the formation of blood clots in blood vessels, and it results in severe complications if clots move to crucial parts of the circulatory system. Thrombotic diseases are major reasons for mortality worldwide [1]. In view of the increasing incidence of thrombotic diseases, effective drugs are urgently desired. The anticoagulant drug represented by heparin has been widely used in the therapy and prophylaxis of thrombosis. However, heparin exhibits some side effects, such as thrombocytopenia, hemorrhagic complications, and congenital or acquired antithrombin deficiency invalidation [2]. Thus, it is necessary to look for alternative sources of anticoagulant agents.

Marine algae are a huge source of natural products, and have gained significant attention for the development of marine drugs due to their structural diversity. Among these, polysaccharides occupy a preeminent position and have high potential as preventative and therapeutic agents [3,4,5,6]. Some investigations have revealed that the sulfated polysaccharides from marine algae are one good source of naturally occurring anticoagulant agents and become an important research area in the search for new anticoagulant drugs [7]. Sulfated galactans and sulfated fucans from red and brown algae are currently the most studied algal sulfated polysaccharides with anticoagulant activity [8,9,10,11]. Heparinoid-active sulfated polysaccharides with different backbones from green algae have also been found [12,13,14]. The sulfated rhamnan from green algae *Monostroma* species exhibited a high anticoagulant activity [15,16]. Hayakawa et al. [17] reported that the sulfated polysaccharides from the genus *Monostroma* had more potent effects on the inhibition of thrombin than heparin. A sulfated galactan from the green alga *Codium divarticum* exhibited a strong anticoagulant activity which was mainly attributed to strong potentiation thrombin by heparin cofactor II (HCII) [18]. Qin et al. [19] found that the sulfated arabinogalactan from the green alga *Chaetomorpha linum* possessed a potent anticoagulant activity in vitro or in vivo, and it was a thrombin inhibitor mediated by antithrombin III (ATIII) or HCII. The sulfated xylogalactoarabinan from *Cladophora falklandica* could modify the kinetics of fibrin formation, reduce the time of permanence of the clot in the blood vessel and induce a less thrombogenic state [20]. However, sulfated polysaccharides from green algae are only partly explored due to their great diversity.

The genus *Chaetomorpha* is one of the largest genera in green algae and is widely distributed throughout the World’s sea. In the present work, an anticoagulant-active sulfated polysaccharide was isolated from the green alga *C. aerea*. The structure of the sulfated polysaccharide was characterized by a combination of chemical and spectroscopic methods and its effects on coagulation factors were studied. 

## 2. Results and Discussion

### 2.1. Structural Characteristics of the Sulfated Polysaccharide PCA

**Figure 1:**
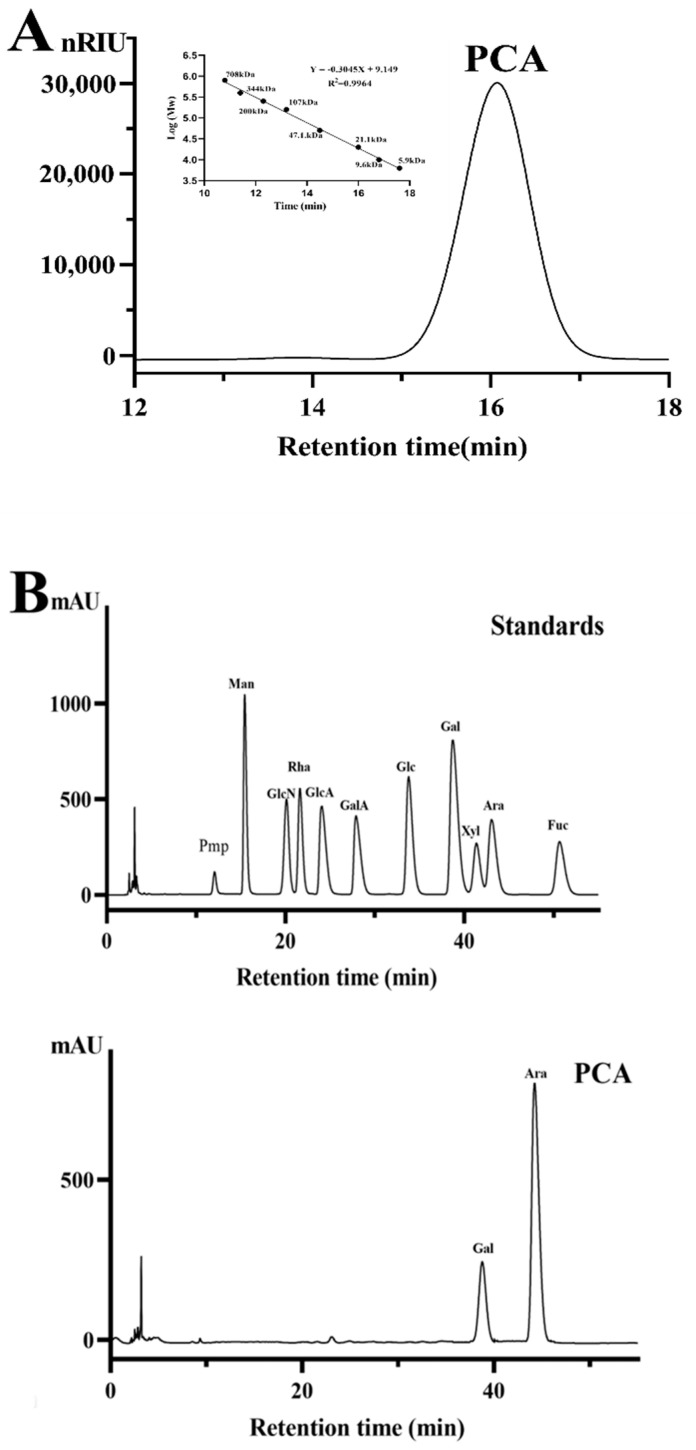

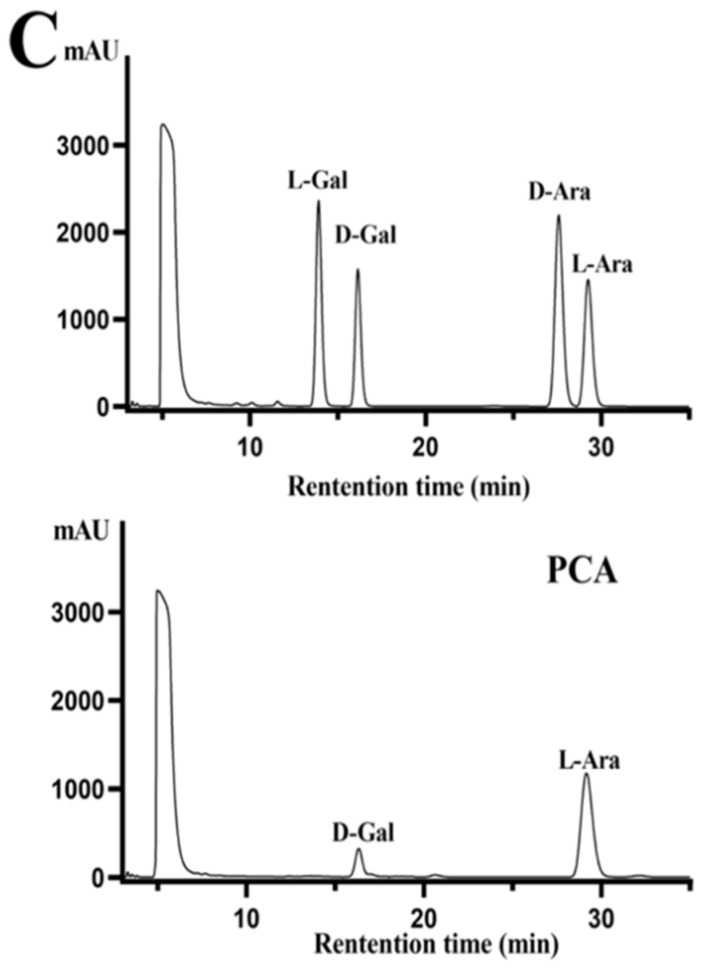
HPGPC and HPLC chromatograms of PCA. (**A**) HPGPC chromatogram and the standard curve of molecular weight; (**B**) HPLC chromatogram of monosaccharide composition assay (Man: d-mannose, GlcN: d-glucosamine, Rha: l-rhamnose, GlcA: d-glucuronic acid, GalA: d-galacturonic acid, Glc: d-glucose, Gal: d-galactose, Xyl: d-xylose, Ara: l-arabinose, Fuc: l-fucose); and (**C**) HPLC chromatogram of sugar configuration determination.

A polysaccharide from *C. aerial,* designated PCA, was obtained by extraction with 0.5 mol/L NaOH, and further purification on Q Sepharose Fast Flow column and Sephacryl S-400/HR column. The yield of PCA from starting algal material was about 1.58% (*w/w*). PCA gave a single and symmetrical peak in the high performance gel permeation chromatography (HPGPC) chromatogram (Figure 1A). The molecular weight of PCA was estimated to be about 18.48 kDa based on its retention time in the HPGPC chromatogram. PCA contained 87.10% total sugar and 22.62% sulfate. No uronic acid and protein were detected in PCA. The UV spectrum analysis of PCA indicated that no absorption peak appeared at 280 nm, further illustrating that PCA did not contain protein. The result of the high performance liquid chromatography (HPLC) assay demonstrated that PCA consisted of arabinose (75.5%) and galactose (24.5%). No other monosaccharide was found in the HPLC chromatogram, also indicating that no uronic acid was present in PCA. The sugar configuration analysis by HPLC indicated that the arabinose in PCA is l-configuration, and the galactose is d-configuration (Figure 1B,C). In order to gain the detailed structural information of PCA, the desulfated product of PCA was prepared. The sulfate content of dsPCA was 0.65%. dsPCA gave a single peak in the HPGPC chromatogram and no peaks appeared in the oligomer region (Figure S1A), indicating that no glycosidic bond was destroyed in the desulfation procedure. The molecular weight of dsPCA was estimated to be about 14.10 kDa. Reversed-phase HPLC analysis demonstrated that the monosaccharide composition of dsPCA was similar to that of PCA (Figure S1B).

To obtain the information of the linkage pattern and sulfate position in PCA, a comparative methylation analysis between PCA and dsPCA was carried out. The results showed that PCA and dsPCA consisted of Ara*p*-(1→, Ara*f*-(1→, →4)-Ara*p*-(1→ (or →5)-Ara*f*-(1→), →3,4)-Ara*p*-(1→, →2,4)-Ara*p*-(1→ (or →2,5)-Ara*f*-(1→), →6)-Gal*p*-(1→, →4)-Gal*p*-(1→ and →4,6)-Gal*p*-(1→ residues (Table 1). Compared with the result of PCA, the amount of →4)-Ara*p*-(1→ residue increased and the →6)-Gal*p*-(1→ residue appeared, whereas the →4,6)-Gal*p*-(1→ and →3,4)-Ara*p*-(1→ residues disappeared in dsPCA. Therefore, the sulfate substitution was deduced to be at the C-4 of →6)-Gal*p*-(1→ and C-3 of →4)-Ara*p*-(1→. The presence of →2,4)-Ara*p*-(1→ illustrated that dsPCA had partial branches at C-2 of →4)-Ara*p*-(1→ residues. Further analysis of NMR spectroscopy was needed to confirm the linkage patterns of the galactose and arabinose.

To further elucidate the structure of PCA, the NMR spectra of dsPCA was analyzed. In the ^1^H NMR spectrum of dsPCA (Figure S2A), six signals of anomeric proton occurred at 4.49, 5.07, 5.13, 5.19, 5.24 and 5.25 ppm. The signals of anomeric carbon appeared at 97.96, 98.28, 105.02, 109.43 and 110.76 ppm in the ^13^C NMR spectrum of dsPCA (Figure S2B). According to the result of methylation analysis, it could be deduced that the anomeric carbon signals at 109.43 and 110.76 ppm were likely signals of α-linked arabinofuranose unit [19]. The ^1^H NMR spin systems of dsPCA were further assigned by the ^1^H–^1^H COSY and ^1^H–^13^C HSQC spectra (Figure S2C,D). The H-1 of A at 4.49 ppm was related to the C-1 at 105.02 ppm, and the H-1 of D at 5.19 ppm was related to the C-1 at 98.28 ppm. The H-4/C-4 of A and H-6/C-6 of D were at 4.20/77.82 ppm and 3.70/65.12 ppm, respectively. Therefore, A was →4)-β-d-Gal*p*-(1→ unit and D was →6)-α-d-Gal*p*-(1→ unit. The H-1 of B at 5.07 ppm was related to the C-1 at 109.43 ppm. B was attributed to →2,5)-α-l-Ara*f*-(1→ residue because of the down-field chemical shifts of C-2 at 84.30 ppm and C-5 at 71.05 ppm. The H-1 of C at 5.13 ppm was correlated to the C-1 at 97.96 ppm. The H-1 of E at 5.24 ppm was related to the C-1 at 110.76 ppm. E was assigned to →5)-α-l-Ara*f*-(1→ residue because of the down-field chemical shifts of C-5 at 71.05 ppm. The H-4/C-of C4 was at 4.10/76.97 ppm, thus C was ascribed to →4)-β-l-Ara*p*-(1→ unit. Compared with →4)-β-l-Ara*p*-(1→ residue, the C-2 shift of F changed to low displacement above 75.86 ppm which represented F and was →2,4)-β-l- Ara*p*-(1→ unit. By combining the data from the ^1^H–^1^H COSY and ^1^H–^13^C HSQC spectra, the assignment of the main proton and carbon signals of the six sugar residues could be completed (Table 2).

**Figure 2:**
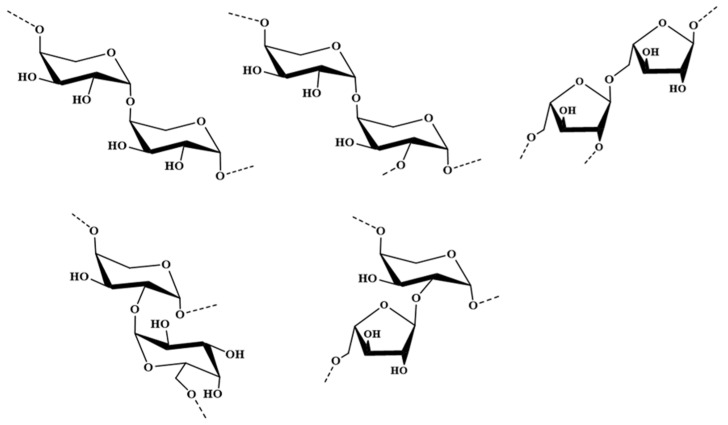
Possible structures of the major disaccharides in dsPCA.

The ^1^H–^13^C HMBC spectrum of dsPCA (Figure S2E) gave the linkage sequences of various glycosidic units. The corresponding signals H-1 (C)/C-4 (C,F) showed the sequences [→4)-β-l-Ara*p*-(1→4)-β-l-Ara*p*-(1→] and [→4)-β-l-Ara*p*-(1→2,4)-β-l-Ara*p*-(1→]. The related signal H-5 (B)/C-1 (E) indicated the linkage [→2,5)-α-l-Ara*f*-(1→5)-α-l-Ara*f*-(1→]. The cross signal H-1 (D)/C-2 (F) confirmed the fragment [→6)-α-d-Gal*p*-(1→2,4)-β-l-Ara*p*-(1→]. Moreover, the related signals H-2 (F)/C-1(E) confirmed the linkage [→5)-α-l-Ara*f*-(1→2,4)-β-l-Ara*p*-(1→]. The possible major disaccharides in dsPCA were listed in Figure 2. 

From the above results, it was concluded that PCA was mainly constituted by a backbone of →4)-β-l-Ara*p*-(1→ units with partial sulfate esters at C-3 of →4)-β-l-Ara*p*-(1→ unit and C-4 of →6)-α-d-Gal*p*-(1→ unit. The side chains consisting of →6)-α-d-Gal*p*-(1→ and →5)-α-l-Ara*f*-(1→ residues were at C-2 of →4)-β-l-Ara*p*-(1→ residue.

Up to now, the report on the structural characteristics of sulfated polysaccharides from the genus *Chaetomorpha* was seldom found. Qin et al. [21] isolated a sulfated rhamnogalactoarabinan from the green alga *C. linum*; its backbone was composed of (1→4) and (1→3,4)-β-l-arabinopyranose with sulfate groups at C-2/C-3 of (1→4)-β-l-arabinopyranose, and the branches contained 4-linked, 6-linked β-d-galactopyranose and terminal rhamnose residues. Recently, arabinan-type sulfated polysaccharides from other green algae were reported. A sulfated heteropolysaccharide from *Cladophora glomerata* Kützing had the backbone of (1→4)-*α*-l-arabinopyranosyl residues with branches and sulfate esters mainly at the C-2 position [22]. He et al. [23] characterized a sulfated galactoarabinan from the green alga *Cladophora oligoclada*, which was constituted by a backbone of (1→4)-β-l-arabinopyranose units with partial sulfate at C-3 of (1→4)-β-l-arabinopyranose unit, and the side chains consisted of (1→4)-β-l-arabinopyranose, (1→4)-β-d-galactopyranose and/or (1→4,6)-β-d-galactopyranose units. The structural characteristics of the sulfated galactoarabinan PCA from *C. aerea* were distinguished from those of the sulfated galactoarabinans previously obtained from green algae. The differences in structure of these sulfated polysaccharides could be derived from the species, eco-physiological growth conditions, biosynthesis machinery, harvest time and extraction methods. The present result suggested that marine green algae could be a potential source of sulfated polysaccharides with novel structures. Further work is required to investigate its structural diversity in relation to its functional properties. 

### 2.2. Anticoagulant Activity In Vitro and In Vivo of PCA

**Figure 3:**
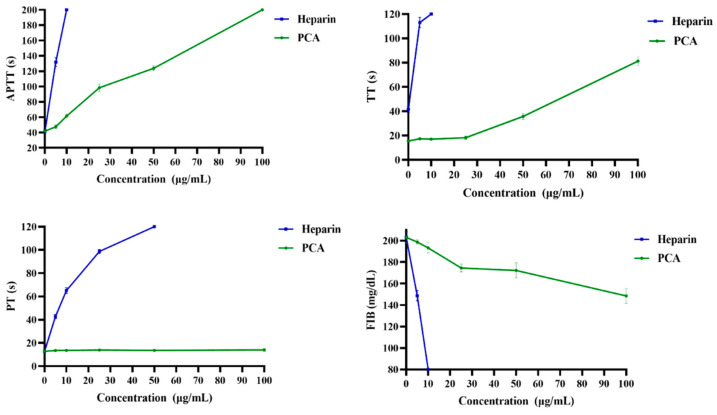
Anticoagulant activity assay in vitro of PCA. Values were mean ± SD (*n* = 3).

Anticoagulant activity in vitro of PCA was evaluated by activated partial thromboplastin time (APTT), thrombin time (TT), prothrombin time (PT) and fibrinogen (FIB) content assays. In the study, heparin was used as a positive control. As shown in Figure 3, PCA significantly prolonged the APTT, and the signal for clotting time was more than 200 s at 100 μg/mL. The prolongation of APTT indicates inhibition of the intrinsic and/or common pathway. TT was also prolonged by PCA, and the clotting time was about 83.7 s at 100 μg/mL. TT reflects the blood coagulation status that transforms fibrinogen into fibrin. APTT and TT activities of PCA were concentration-dependent. It was noted that the prolongation effect of PCA on the APTT activity was stronger than that on the TT activity. The anticoagulant activity of PCA was weaker than that of heparin, and higher concentrations were required to achieve the same effect as with heparin in the APTT and TT assays. In addition, the effect of PCA on PT was markedly different from that of heparin. PCA had no apparent clotting inhibition to PT, even at the concentration at which APTT and TT were prolonged. No prolongation of PT demonstrates no inhibition of the extrinsic pathway of coagulation. Furthermore, PCA effectively decreased the fibrinogen level in a concentration-dependent manner. Fibrinogen is a key factor in coagulation. High fibrinogen levels can avoid platelet aggregation, and thus prevent the formation of hypercoagulable states and thrombosis. These data illustrated that PCA had a strong anticoagulant activity in vitro which inhibited both the intrinsic and/or common pathways of coagulation, and thrombin activity or conversion of fibrinogen to fibrin. 

**Figure 4:**
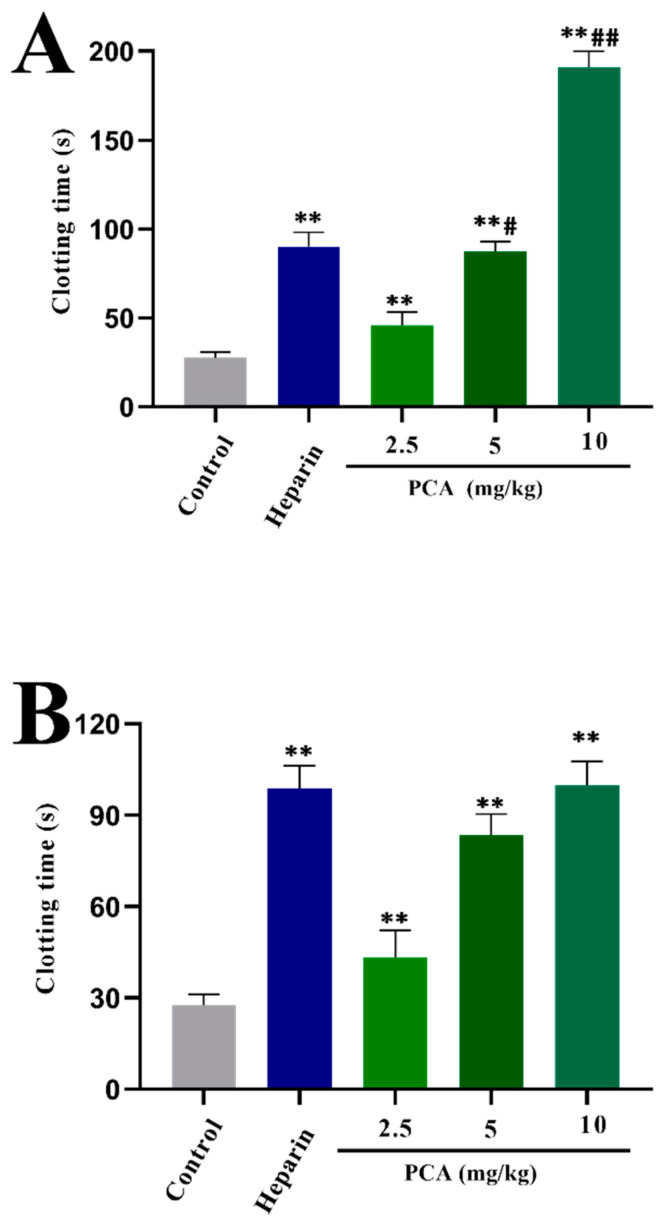
Anticoagulant activity assay in vivo of PCA. (**A**) APTT; and (**B**) TT. Values were mean ± SD (*n* = 3). Significance: ** *p* < 0.01 vs. the control group; # *p* < 0.05 vs. the heparin group, ## *p* < 0.01 vs. the heparin group.

Anticoagulant activity of PCA in vivo was further evaluated by assays of APTT, PT and TT. After intravenous injection of heparin and PCA, no rats were found moribund and bleeding. Moreover, the clotting times were prolonged after treatment with PCA and heparin; the results illustrated that PCA and heparin were absorbed. At the dose of 2.5 or 5 mg/kg, PCA significantly prolonged the APTT and TT (Figure 4), but it had no prolongation effect on the PT (data not shown). The anticoagulant activity in vivo of PCA was concentration-dependent. Furthermore, the APTT activity of PCA at 10 mg/kg was higher than that of heparin at the concentration used in the experiment. The prolongation effect of PCA at 10 mg/kg on the TT activity is close to that of heparin at the concentration used. These data indicated that PCA possessed a strong anticoagulant activity in vivo and could be a promising source of anticoagulant agent. 

The sulfated polysaccharide PCA showed similar anticoagulant activity with the sulfated polysaccharide CLS4 from *C. linum* which was effective in prolongation of APTT and TT, but poor in modulating PT [19]. CLS4 was a sulfated arabinogalactan which consisted of (1→6)-β-d-galactopyranose and (1→5)-α-l-arabinofuranose residues with sulfate groups at C-2/C-3 of (1→5)-α-l-arabinofuranose and C-2/C-4 of (1→6)-β-d-galactopyranose. In addition, the ability of PCA to stimulate prolongation of APTT or TT differed from that of the sulfated xylogalactoarabinan from *Cladophora falklandica* [20], although they were arabinan-type sulfated polysaccharides. The prolongation effect of the sulfated xylogalactoarabinan on the APTT was weaker than that on the TT, whereas the APTT increase in PCA was higher than that observed in TT. The differences in anticoagulant properties may be attributed to the structural variation. An in-depth investigation on the anticoagulant activity of sulfated polysaccharides with different structures will indubitably play an important role in the understanding of the anticoagulant property.

### 2.3. Effects of PCA on the Coagulation Factors II, V, VIII, X, IX, XI and XII 

**Figure 5:**
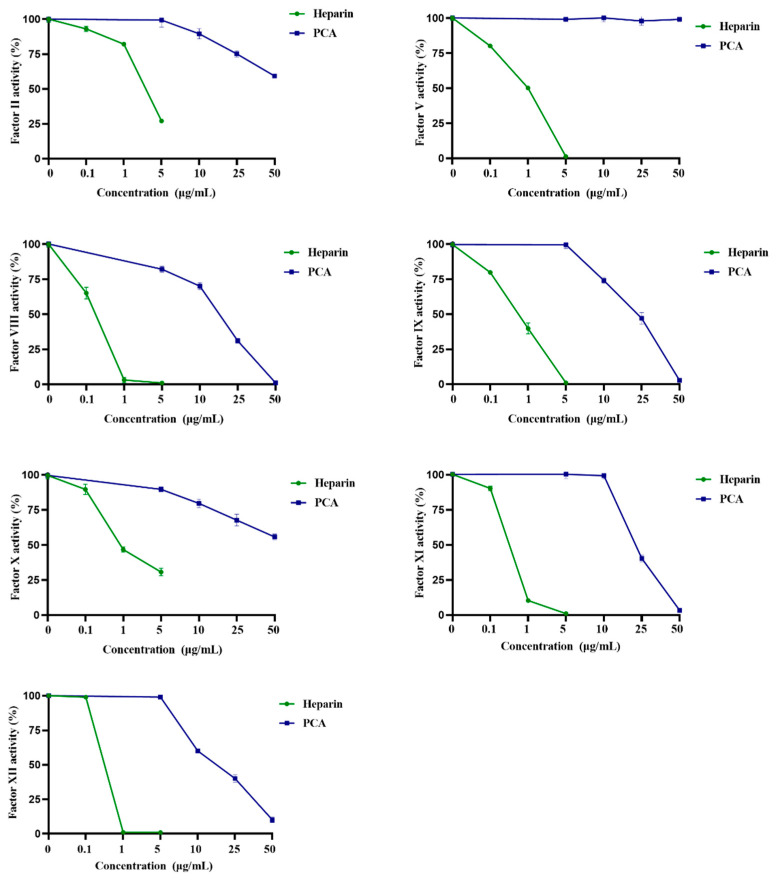
Effects of PCA on the coagulation factors II, V, VIII, IX, X, XI and XII. Values were mean ± SD (*n* = 3). The activity of coagulation factor was estimated by reference to calibration curves made by serial dilutions of standard human plasma. The standard human plasma with a dilution ratio of 1:5 (plasma/imidazole buffer, *v/v*) was used as a control. The mean value of coagulation factor activity of control was defined as 100%.

The blood coagulation system consists of intrinsic and extrinsic pathways, where a series of factors are involved in the mechanism. As endogenous or exogenous anticoagulants interfere with the coagulation factors by inactivating or restricting them, the blood coagulation can be prolonged or stopped [3]. The coagulant factors associated with the intrinsic pathway involve XII, XI, IX and VIII. For the common pathway, coagulant factors include II, V and X [19]. The effects of PCA on the coagulation factors were evaluated using heparin as a reference. As shown in Figure 5, the activities of the coagulation factors XII, XI, IX and VIII were effectively inhibited by PCA in a concentration-dependent manner. Moreover, the inhibition ability of PCA on coagulation factor VIII was higher than those on the factors XII, XI and IX. In addition, PCA also possessed inhibition effects on the common pathway factors II and X. However, the inhibitory effects of PCA on common pathway factors II and X were obviously weaker than those on intrinsic pathway factors XII, XI, IX and VIII. PCA did not have a significant inhibition effect on coagulation factor V. The results demonstrated that PCA inhibited all the intrinsic coagulation factors, but partially inhibited the common coagulation factors.

Until now, a few studies in the literature were correlated to the effects of algal sulfated polysaccharides on coagulation factors. He et al. [23] reported that the sulfated polysaccharide OCSH4 from the green alga *Cladophora oligoclada* largely inhibited all the intrinsic coagulation factors, selectively inhibited the common coagulation factors and it did not have a significant effect on the factor X. Qin et al. [19] also found that the sulfated polysaccharide CLS4 from the green alga *C. linum* significantly inhibited the activities of the coagulation factors XII, XI, IX and VIII, and the inhibition effects of CLS4 on the coagulation factors XII and XI were stronger than those on the factors IX and VIII. The ability of PCA to inhibit the activities of the coagulation factors differed from those of the sulfated polysaccharides from *Cladophora oligoclada* and *C. linum.* The differences in the inhibition effect on coagulation factors may be associated with the structure of the sulfated polysaccharides, such as monosaccharide composition, sulfation pattern and glycosidic linkage. The inhibition of coagulation factors XII, XI, IX and VIII could be effective for the health promotion and treatment of cardiovascular disease [24]. The present results indicated that PCA could be a potential anticoagulant for prevention and therapy of thrombotic diseases. 

### 2.4. Effects of PCA on Thrombin and Factor Xa Inhibition Mediated by HCII or ATIII

**Figure 6:**
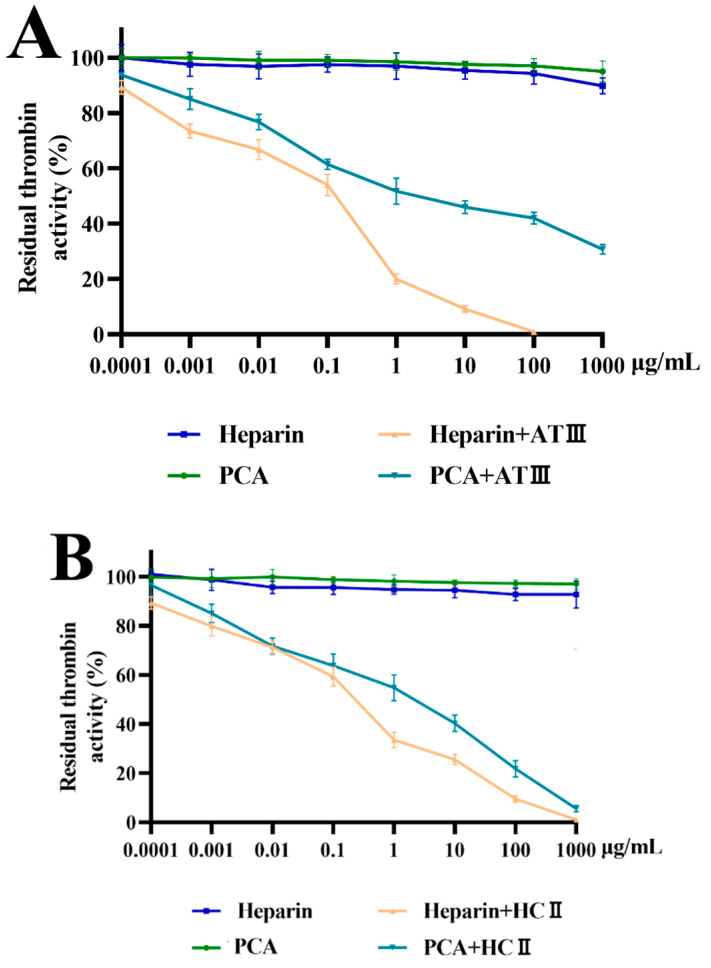

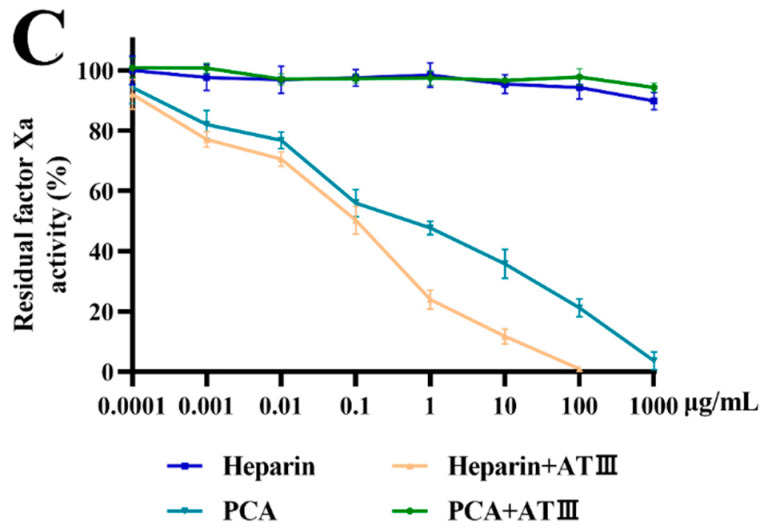
Effects of PCA on thrombin and factor Xa activity mediated by HCII or ATIII. Values were mean ± SD (*n* = 3). (**A**) inhibition of thrombin by ATIII; (**B**) inhibition of thrombin by HCII; and (**C**) inhibition of factor Xa by ATIII.

To elucidate the anticoagulant mechanism of PCA, its inhibition effects on thrombin and coagulation factor Xa were studied using chromogenic substrate in the absence and in the presence of ATIII or HCII. Normally, ATIII inhibits all intrinsic pathway coagulation enzymes, and HC-II is a serine protease inhibitor and selectively inhibits thrombin. As shown in Figure 6A,B, PCA had no apparent effect on thrombin inhibition in the absence of HCII or ATIII. However, PCA exhibited a significant effect on thrombin inhibition mediated by ATIII in a dose-dependent manner (Figure 6A). Additionally, a strong effect of PCA on HCII-dependent thrombin inhibition was also found (Figure 6B). However, the ability of PCA to inhibit thrombin in the presence of HCII or ATIII was lower than that of heparin. When using FXa instead of thrombin as the target protease (Figure 6C), the amidolytic activity of FXa was also not directly inhibited by PCA in the absence of ATIII. PCA exhibited a strong effect on the amidolytic activity of FXa in an ATIII-dependent pathway. In a similar fashion, if ATIII was replaced by HCII as the target plasma inhibitor, the effect of PCA on HCII-dependent FXa inhibition was not observed (data not shown). The results demonstrated that PCA was a potent thrombin inhibitor mediated by HCII or ATIII and it also hastened factor Xa inhibition potentiated by ATIII. 

Little work was associated with the anticoagulant mechanism of sulfated polysaccharides from green algae. The inhibition of thrombin by PCA was different from that of the arabinan-type sulfated polysaccharides from the green algae *Codium vermilara* and *Cladophora falklandica* which had direct thrombin interaction [20,25]. In addition, PCA had obvious differences in thrombin and factor Xa inhibition mediated by ATIII or HCII compared with the sulfated rhamnan from green algae *Monostroma* species [16]. The sulfated rhamnan had no inhibitory effect on factor Xa in the presence of ATIII and it possessed a stronger thrombin inhibition than heparin. It was also noted that the inhibition effects of PCA on thrombin and factor Xa by potentiating ATIII or HCII were similar to those of the sulfated polysaccharide from *C. linum* [19], although the latter was mainly composed of galactose. The results indicated that complex relationships existed between the structure and anticoagulant property of the sulfated polysaccharides [7]. Further investigation of the anticoagulant mechanism of the algal sulfated polysaccharides is required. 

Anticoagulant-active polysaccharides from marine algae hold great promise as a potential alternative source of heparin from animal sources for therapy. Presently, the research on anticoagulant activity of sulfated polysaccharides from green algae is still less comprehensive than that on sulfated polysaccharides from brown and red algae [26,27]. The great diversity and metabolite complexity of green algae offer a unique and exclusive source of renewable drug molecules. Sulfated polysaccharides with novel structure from green algae have good anticoagulant activity and their anticoagulant actions are even stronger than heparin in some respects [19,28]. It is crucial to obtain more comprehensive information on the anticoagulant activity of algal sulfated polysaccharides, including the inhibition of coagulation factors. Continuous works will promote the development of anticoagulant agents from marine algae. 

## 3. Materials and Methods

### 3.1. Materials

*C. aerea* was collected from Yantai, China on May 2017. The raw material was thoroughly washed with tap water, air-dried, milled and stored at room temperature in a dry environment. Q Sepharose Fast Flow and Sephacryl S-400/HR were from GE Healthcare Life Sciences (Piscataway, NJ, USA). Dialysis tubing (cellulose membrane; flat width 44 mm, molecular weight cut-off 3500 Da) was from Lvniao (Yantai, China). Pullulan standards (*Mw*: 5.9, 9.6, 21.1, 47.1, 107, 200, 344 and 708 kDa) were from Showa Denko K.K. (Tokyo, Japan). Heparin (196 U/mg) was from Sigma (St. Louis, MO, USA). APTT, TT, PT and FIB kits were from MD Pacific (Tianjin, China). Standard human plasma and a series of deficient human plasma (lacking factor XII, XI, IX, VIII, II, V or X) were from Siemens (Munich, Germany). Factor Xa and thrombin were from Boatman Biotech CO., LTD. (Shanghai, China). ATIII was from Chromogenix (Milan, Italy). HCII was from Hyphen Biomed (Neuville, France). Chromogenic substrates S-2238 and S-2765 were from Asnail (Beijing, China).

### 3.2. Isolation and Purification of the Sulfated Polysaccharide PCA

The milled alga (120 g) was defatted with 95% ethanol (*v/v*) for 4 h. After removing ethanol, the alga was extracted using a 40-fold volume of distilled water at 100 °C for 4 h, then cooled and centrifugated. The remaining biomass was then dipped into 4 L of 0.5 mol/L NaOH and extracted at room temperature for 4 h. After centrifugation (5000× *g*, 10 min), the supernatant was concentrated and dialyzed in a cellulose membrane (molecular weight cut-off 14 kDa) against flowing distilled water at room temperature. The recovered fraction was concentrated and precipitated by adding a 4-fold volume of 95% ethanol (*v/v*). The precipitate recovered by centrifugation was dried at 40 °C to yield the crude polysaccharide (5.65 g). The crude polysaccharide was separated by a Q Sepharose Fast Flow column (60 × 3.5 cm) eluted with a step-wise gradient of 0, 0.5, 1.0 and 1.5 mol/L NaCl. Subsequently, the fraction eluted with 1.5 mol/L NaCl was further purified on a Sephacryl S-400/HR column (100 × 2.5 cm) and eluted with 0.2 mol/ L NH_4_HCO_3_ at a flow rate of 0.3 mL/min. The major polysaccharide fractions were pooled, desalted, freeze-dried and designated PCA (1.89 g).

### 3.3. Assay of Physicochemical Property 

Total sugar content was measured by the phenol-sulfuric acid method [29]. Protein content was determined according to the method of Bradford [30]. Sulfate content was assessed by the barium rhodizonic acid method [31]. Purity and molecular weight of polysaccharide were assessed by HPGPC on a Shodex OHpak SB-804 HQ column [19]. The molecular weight was estimated by reference to a calibration curve made by pullulan standards. Monosaccharide composition was assayed by a 1-phenyl-3-methyl-5-pyrazolone (PMP)-HPLC method using the Eclipse XDB-C18 column (4.6 × 250 mm, Agilent Technologies, Santa Clara, CA, USA) [21]. Sugar configuration was determined by reversed-phase HPLC [32] and the analysis was performed on an Agilent 1260 Infinity HPLC instrument (Agilent Technologies, Santa Clara, CA, USA) using an Agilent XDB-UV detector (250 nm).

### 3.4. Desulfation

Desulfation of PCA was performed according to Miller and Blunt [33]. PCA (100 mg) was dissolved in water and passed through a 732 cation-exchange resin column (H+ form), which was eluted with distilled water. The combined effluent was adjusted to pH 9.0 with pyridine and then lyophilized to give a white powdered pyridinium salt. The product was dissolved in 6 mL of dimethyl sulfoxide containing 65 mg of pyromellitic acid and 60 mg of NaF, and then 2 mL of pyridine was added. The solution was shaken at 100 °C for 4 h. After the reaction was completed, the product was dialyzed against distilled water, freeze-dried and named as dsPCA. 

### 3.5. Methylation Analysis

Methylation analyses of PCA and dsPCA were carried out according to the method of Harris et al. [34]. Briefly, polysaccharide in dimethyl sulfoxide was methylated using NaH and iodomethane. After hydrolysis with 2 mol/L trifluoroacetic acid at 105 °C for 6 h, the methylated sugar residues were converted to partially methylated alditol acetates by reduction with NaBH_4_, followed by acetylation with acetic anhydride. Thereafter, partial methylated alditol acetates were analyzed on a TRACE 1300-ISO GC–MS spectrometer (Thermo Scientific, Waltham, MA, USA) fitted with a DB 225 fused silica capillary column.

### 3.6. NMR Spectroscopy Analysis

1D and 2D NMR spectra were recorded at 25 °C on an Agilent DD2 500 MHz NMR spectrometer (Agilent Technologies, Santa Clara, CA, USA). Polysaccharide (50 mg) was deuterium-exchanged by lyophilization two times with 99% D_2_O and then was dissolved in 1.0 mL of 99.97% D_2_O. Acetone (^1^H: 2.225 ppm, ^13^C: 31.07 ppm) was taken as the internal standard. Spectra were processed and analyzed using MestReNova (V12.0.3, Mestrelab Research, Santiago de Compostela, Spain).

### 3.7. Analysis of Anticoagulant Activity In Vitro

APTT, TT, PT and FIB assays were carried out as described previously [35,36]. Polysaccharide (5, 10, 25, 50, or 100 μg/mL) was dissolved in 0.9% NaCl. For APTT assay, 90 μL of citrated human plasma was mixed with 10 μL of polysaccharide solution and cultured at 37 °C for 1 min. A total of 100 μL of prewarmed APTT reagent was added and cultured at 37 °C for 2 min. Thereafter, 100 μL of pre-warmed CaCl_2_ (0.025 mol/L) was added and clotting time was measured in a SL318 coagulometer (Senlan Medical Science and Trading CO., LTD., Jinan, China). For PT assay, 90 μL of citrated human plasma was mixed with 10 μL of polysaccharide solution and cultured at 37 °C for 1 min. Then, 200 μL of pre-warmed PT assay reagent was added and clotting time was measured. For TT assay, 90 μL of citrated human plasma was mixed with 10 μL of polysaccharide solution and cultured at 37 °C for 1 min. Then, 200 μL of prewarmed TT assay reagent was added and clotting time was determined. For FIB content assay, 90 μL of citrated human plasma was mixed with 10 μL of polysaccharide solution, then was diluted with 900 μL of imidazole buffer. Next, 200 μL of the diluted plasma sample was taken and cultured at 37 °C for 3 min, followed by the addition of 100 μL of FIB assay reagent, and then clotting time was measured. The FIB content was estimated by reference to a calibration curve made by FIB constant value plasma. Heparin was used as a positive control. Saline solution (0.9% NaCl) was used as a control.

### 3.8. Animals

Male Sprague-Dawley rats (180–220 g body weight) were housed at 23 ± 2 °C under a 12 h light/dark cycle with free access to food and water. All animal experiments were approved by the Institutional Animal Care and Use Committee of the Ocean University of China (OUC-YY-201901001).

### 3.9. Determination of Anticoagulant Activity In Vivo

Anticoagulant activity in vivo was performed by assays of APTT, TT and PT using rat plasma. Briefly, Sprague-Dawley rats were randomly divided into six experimental groups (6 rats/group). The experimental rats were anaesthetized with 15% urethane, then injected with PCA (2.5, 5, 10 mg/kg) and heparin (0.5 mg/kg). Saline solution (0.9% NaCl) was used as a control. After 30 min, to allow for circulation, the rats were secured in the supine position and the blood was drawn from the abdominal aorta. APTT, TT and PT assays of the rat plasma were performed using commercial kits according to abovementioned methods. 

### 3.10. Assay of Coagulation Factor II, V, X, VIII, IX, XI, or XII Activity

The assay was performed according to the method of Takahashi and Hiraga [37] using standard human plasma and deficient human plasma. Polysaccharide (5, 10, 25, 50, or 100 μg/mL) was dissolved in imidazole buffer (pH 7.3). For factor XII, XI, IX or VIII activity assay, 90 μL of re-dissolved standard human plasma was mixed with 10 μL of polysaccharide solution, 100 μL of deficient human plasma (lacking factor XII, XI, IX or VIII) and 100 μL of APTT reagent were added, and cultured at 37 °C for 2 min. Then, pre-warmed 0.025 mol/L CaCl_2_ (100 μL) was added and clotting time was measured. For factor II, V or X activity assay, 90 μL of re-dissolved standard human plasma was mixed with 10 μL of polysaccharide solution, 100 μL of deficient human plasma (lacking factor II, V or X) was added and cultured at 37 °C for 1 min. Thereafter, 100 μL of pre-warmed PT assay reagent was added and clotting time was determined. The activities of coagulation factors (II, V, VIII, IX, X, XI and XII) were assayed by reference to calibration curves made by serial dilutions of standard human plasma. Standard human plasma was diluted with imidazole buffer. The standard human plasma with a dilution ratio of 1:5 (plasma/imidazole buffer, *v/v*) was used as a control. The mean value of coagulation factor activity of control was defined as 100%. Heparin was used as a positive control.

### 3.11. Effects of PCA on Thrombin and Factor Xa Inhibition Mediated by ATIII or HCII

The assays were performed as described previously [38]. Human thrombin or coagulation factor Xa and inhibitors (HCII or ATIII) were cultured with or without PCA in 180 μL of 0.02 mol/L trisaminomethane (Tris)−HCl, 0.15 mol/L NaCl and 1.0 mol/L polyethylene glycol at 37 °C. After incubation for 2 min, 20 μL of Tris−HCl buffer containing 1.5 mmol/L chromogenic substrate S-2238 for thrombin or S-2765 for coagulation factor Xa was added, and the residual thrombin or coagulation factor Xa activity was assayed by determining the change in absorbance (405 nm). The change rate of absorbance was proportional to the thrombin or coagulation factor Xa activity remaining in the incubation. Heparin was used as a positive control and saline solution (0.9% NaCl) was used as a control.

### 3.12. Statistical Analysis

The data were presented as mean ± standard deviation (SD). Statistical significance was obtained using Graph Pad Prism 9.2.0 software by a one-way ANOVA test. *p* < 0.05 was regarded as statistically significant.

## 4. Conclusions

The sulfated polysaccharide PCA from *C. aerea* was constituted by a main chain of (1→4)-β-l-Ara*p* units, partially sulfated at C-3 and branched at C-2. The side chains consisted of →6)-α-d-Gal*p* (1→ and →5)-α-l-Ara*f*-(1→. PCA showed a potent anticoagulant activity in vitro and in vivo, and exerted strong inhibition effects on the coagulation factors XII, XI, IX and VIII. The anticoagulation property of PCA was attributed to strong thrombin inhibition mediated by HCII or ATIII, and it also hastened the inhibition of coagulation factor Xa by potentiating ATIII. PCA has the potential to be developed into a novel anticoagulant agent for prevention and therapy of thrombotic diseases. An in-depth investigation on the anticoagulant property of PCA in vivo is underway. Further study on the structure–activity relationship of the sulfated polysaccharides will aid the understanding of their anticoagulant activities and may ultimately lead to the development of novel anticoagulant agents. 

## Figures and Tables

**Table 1:** Methylation analysis results of PCA and dsPCA.

Methylated Alditol Acetate	Molar Percent Ratio		Linkage Pattern
	PCA	dsPCA	
1,5-Di-acetyl-2,3,4-tri-O-methyl-arabinitol	3.96	3.08	Ara*p*-(1→
1,4-Di-acetyl-2,3,5-tri-O-methyl-arabinitol	6.18	6.13	Ara*f*-(1→
1,4,5-Tri-acetyl-2,3-di-O-methyl-arabinitol	28.21	50.98	→4)-Ara*p*-(1→/→5)-Ara*f*-(1→
1,3,4,5-Tetra-acetyl-2-O-methyl-arabinitol	24.00	- ^a^	→3,4)-Ara*p*-(1→
1,2,4,5-Tetra-acetyl-3-O-methyl-arabinitol	10.77	10.89	→2,4)-Ara*p*-(1→/→2,5)-Ara*f*-(1→)
1,5,6-Tri-acetyl-2,3, 4-tri-O-methyl-galactitol	- ^a^	16.98	→6)-Gal*p*-(1→
1,4,5-Tri-acetyl-2,3, 6-tri-O-methyl-galactitol	10.81	11.00	→4)-Gal*p*-(1→
1,4,5,6-Tetra-acetyl-2,3-di-O-methyl-galactitol	16.88	- ^a^	→4,6)-Gal*p*-(1→

^a^ Not detected.

**Table 2:** Signal assignments of NMR spectra of dsPCA.

Residues		Chemical Shifts (ppm) ^a^					
		H1/C1	H2/C2	H3/C3	H4/C4	H5/C5	H6/C6
A	→4)-β-d-Gal*p*-(1→	4.49/105.02	3.38/74.70	3.83/69.95	4.20/77.82	3.86/72.38	3.80/62.10
B	→2,5)-α-l-Ara*f*-(1→	5.07/109.43	4.09/84.30	4.08/78.74	4.10/82.57	3.92/71.05	
C	→4)-β-l-Ara*p*-(1→	5.13/97.96	4.01/69.99	4.14/69.89	4.10/76.97	3.85/61.60	
D	→6)-α-d-Gal*p*-(1→	5.19/98.28	4.19/72.32	3.68/72.46	4.03/69.52	3.89/69.75	3.70/65.12
E	→5)-α-l-Ara*f*-(1→	5.24/110.76	4.50/82.28	4.08/78.74	4.20/82.84	3.92/71.05	
F	→2,4)-β-l-Ara*p*-(1→	5.25/97.96	4.01/75.86	4.25/68.38	4.10/76.97	3.85/61.09	

**^a^** Spectra were performed on an Agilent DD2 500M NMR spectrometer. Chemical shifts are referenced to internal acetone at 2.225 ppm for ^1^H and 31.07 ppm for ^13^C. Gal*p*: galactopyranose, Ara*f*: arabinofuranose, Ara*p*: arabinopyranose.

## Data Availability

Data presented in this study are available on request.

## Supplementary Materials

The following are available online at https://www.mdpi.com/article/10.3390/md21020088/s1, Figure S1: HPGPC and HPLC chromatograms of dsPCA, Figure S2: NMR spectra of dsPCA.
