# Supplementary material for: Anticoagulant Property of a Sulfated Polysaccharide with Unique Structural Characteristics from the Green Alga Chaetomorpha aerea
Source: Mar Drugs. 2023 Jan 26;21(2):88. doi: 10.3390/md21020088 (PMC9962809; doi:10.3390/md21020088)
Supplement: Supplementary file 1 [file marinedrugs-21-00088-s001.zip › marinedrugs-2128639-supplementary.pdf]

Supplementary material for:

Anticoagulant Property of a Sulfated Polysaccharide with Unique Structural Characteristics from the Green Alga *Chaetomorpha aerea*

Ling Qin[1], Yajing Yang[1], Wenjun Mao[1,2,*]

[1] Key Laboratory of Marine Drugs of Ministry of Education, Shandong Provincial Key Laboratory of Glycoscience and Glycotechnology, School of Medicine and Pharmacy, Ocean University of China, Qingdao 266003, China
[2] Laboratory for Marine Drugs and Bioproducts, National Laboratory for Marine Science and Technology (Qingdao), Qingdao 266237, China
* Correspondence: wenjunm@ouc.edu.cn; Tel.: +86-532-8203-1560

SUPPLEMENTARY FIGURES

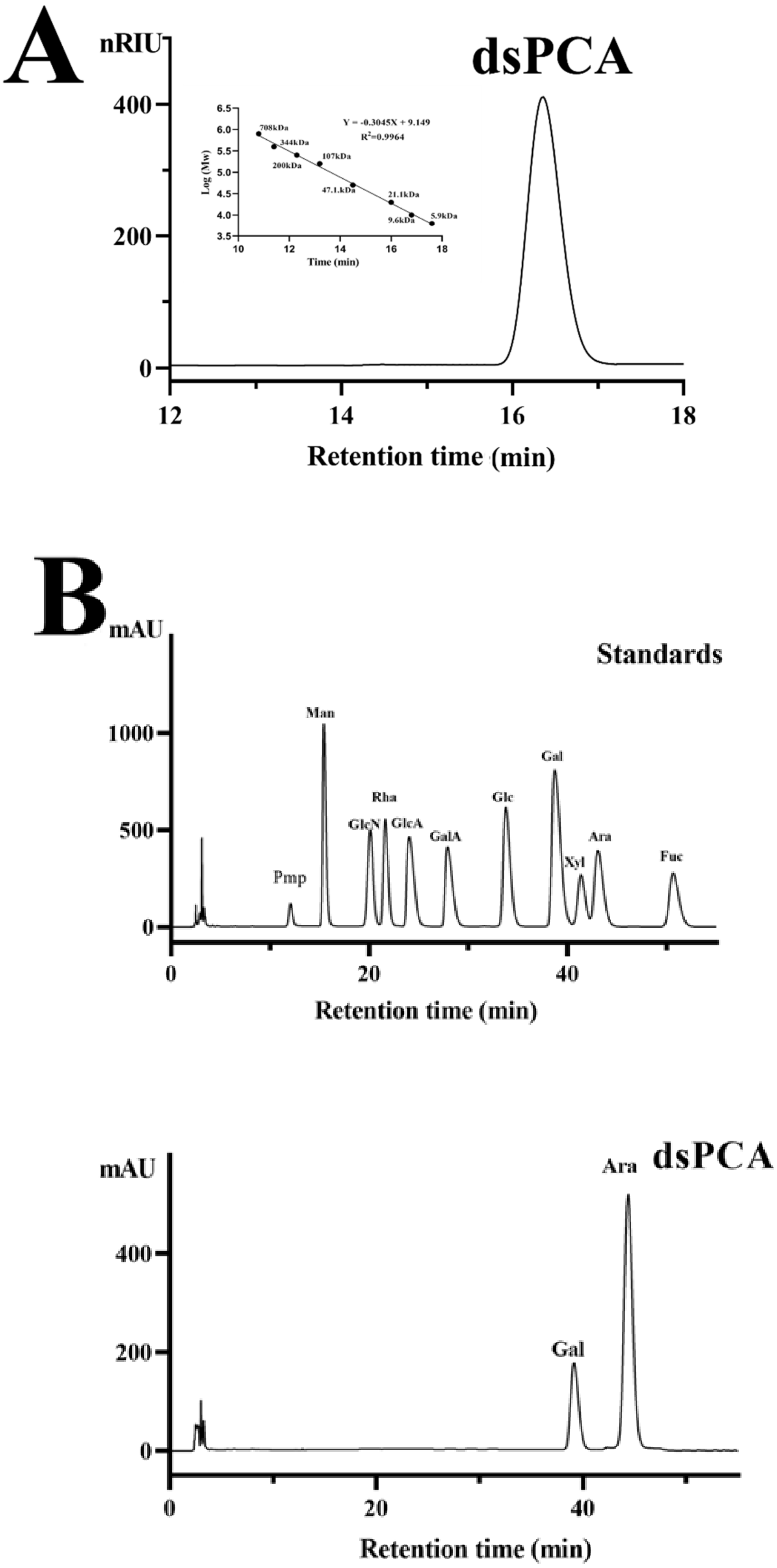

Figure S1. HPGPC and HPLC chromatograms of dsPCA. **(A)** HPGPC chromatogram of dsPCA on a Shodex OHpak SB-804 HQ column and the standard curve of molecular weight; **(B)** HPLC chromatogram for monosaccharide component analysis of dsPCA (Man: D-mannose, GlcN: D-glucosamine, Rha: L-rhamnose, GlcA: D-glucuronic acid, GalA: D-galacturonic acid, Glc: D-glucose, Gal: D-galactose, Xyl: D-xylose, Ara: L-

arabinose, Fuc: L-fucose).

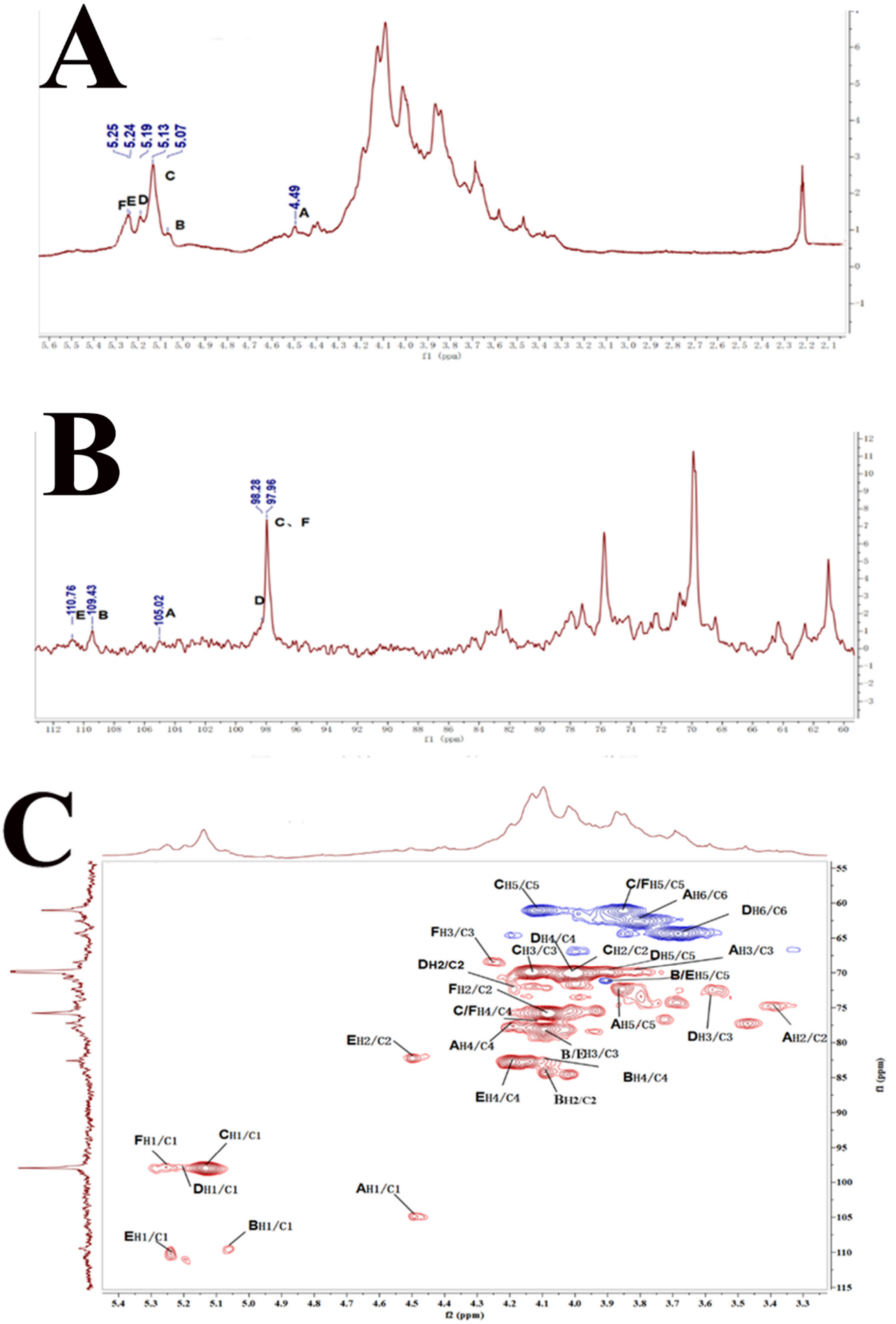

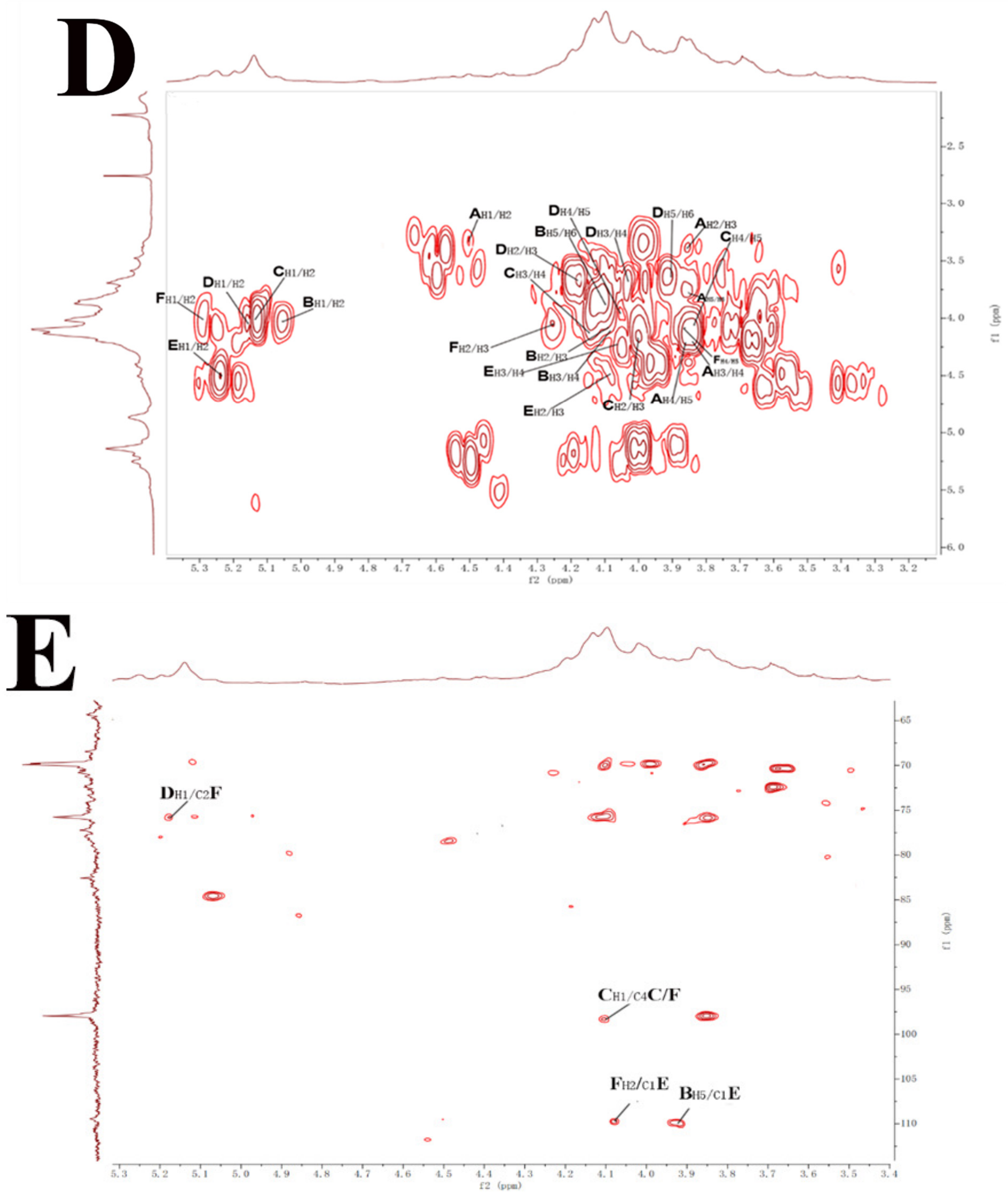

Figure S2. NMR spectra of dsPCA. Spectra were performed on an Agilent DD2 500 MHz NMR spectrometer using acetone as internal standard. **(A)** ^{1}H NMR spectrum; **(B)** ^{13}C NMR spectrum; **(C)** ^{1}H–^{1}H COSY spectrum; **(D)** ^{1}H–^{13}C HSQC spectrum; **(E)** ^{1}H–^{13}C HMBC spectrum. A: →4)-β-D-Gal*p*-(1→ ; B: →2,5)-*α*-L-Ara*f*-(1→; C: → 4)-β-L-Ara*p*-(1 → ; D: → 6)-*α*-D-Gal*p*-(1 → ; E: → 5)-*α*-L-Ara*f*-(1 → ; F: → 2,4)-β-L-Ara*p*-(1 → Gal*p*: galactopyranos, Ara*f*: arabinofuranose, Ara*p*: arabinopyranos.
